# Immunophenotype and function of circulating myeloid derived suppressor cells in COVID-19 patients

**DOI:** 10.1038/s41598-022-26943-z

**Published:** 2022-12-29

**Authors:** Fatemeh Kiaee, Hamidreza Jamaati, Heshmat Shahi, Neda Dalil Roofchayee, Mohammad Varahram, Gert Folkerts, Johan Garssen, Ian M. Adcock, Esmaeil Mortaz

**Affiliations:** 1 Department of Immunology, School of Medicine, Shahid Beheshti University of Medical Sciences, Tehran, Iran; 2 Chronic Respiratory Diseases Research Center, National Research Institute of Tuberculosis and Lung Diseases (NRITLD), Shahid Beheshti University of Medical Sciences, Tehran, Iran; 3 Mycobacteriology Research Center, National Research Institute of Tuberculosis and Lung Diseases (NRITLD), Shahid Beheshti University of Medical Sciences, Tehran, Iran; 4 Division of Pharmacology, Faculty of Science, Utrecht Institute for Pharmaceutical Sciences, Utrecht University, Utrecht, The Netherlands; 5 Priority Research Centre for Asthma and Respiratory Disease, Hunter Medical Research Institute, University of Newcastle, Newcastle, NSW Australia; 6 Airways Disease, National Heart and Lung Institute, Imperial College London, London, UK

**Keywords:** Cell biology, Immunology, Molecular biology

## Abstract

The pathogenesis of coronavirus disease 2019 (COVID-19) is not fully elucidated. COVID-19 is due to severe acute respiratory syndrome coronavirus 2 (SARS-CoV-2) which causes severe illness and death in some people by causing immune dysregulation and blood T cell depletion. Increased numbers of myeloid-derived suppressor cells (MDSCs) play a diverse role in the pathogenesis of many infections and cancers but their function in COVID-19 remains unclear. To evaluate the function of MDSCs in relation with the severity of COVID-19. 26 PCR-confirmed COVID-19 patients including 12 moderate and 14 severe patients along with 11 healthy age- and sex-matched controls were enrolled. 10 ml whole blood was harvested for cell isolation, immunophenotyping and stimulation. The immunophenotype of MDSCs by flow cytometry and T cells proliferation in the presence of MDSCs was evaluated. Serum TGF-β was assessed by ELISA. High percentages of M-MDSCs in males and of P-MDSCs in female patients were found in severe and moderate affected patients. Isolated MDSCs of COVID-19 patients suppressed the proliferation and intracellular levels of IFN-γ in T cells despite significant suppression of T regulatory cells but up-regulation of precursor regulatory T cells. Serum analysis shows increased levels of TGF-β in severe patients compared to moderate and control subjects (HC) (P = 0.003, P < 0.0001, respectively). The frequency of MDSCs in blood shows higher frequency among both moderate and severe patients and may be considered as a predictive factor for disease severity. MDSCs may suppress T cell proliferation by releasing TGF-β.

## Introduction

In December 2019, SARS-CoV-2 infection resulted in a global pandemic termed coronavirus disease 2019 (COVID-19)^1^. The illness is characterized by influenza-like symptoms including fever, cough, and myalgia, and most patients demonstrate mild or moderate symptoms although 19% of patients suffer from severe or critical illness with acute respiratory distress syndrome (ARDS) and even with death^2^. The immunopathogenesis of COVID-19 disease remains unclear and additional fundamental research is required to obtain a better understanding of the disease to enable its control and management.

The systemic blood hallmark of COVID-19 disease is T cell lymphopenia, especially in critical cases^3^, while SARS-CoV-2–specific T cells are important in combating and handling the spread of the virus^4^. During the suppression of T cell responses, monocytes become inactive^5^ which is associated with reduced monocyte HLA-DR expression on myeloid-derived suppressor cells (MDSCs)^6^. MDSCs originate from the myeloid cell lineage and are a diverse group of relatively immature myeloid cells^7,8^. They suppress immune responses, especially T cells responses, in different conditions such as tumors^9–13^. A dysregulation of these cells has been reported in COVID-19 patients^14,15^.

Two subpopulations of MDSCs are recognized due to their morphology, density, and phenotype: monocytic MDSCs (M-MDSCs) and polymorphonuclear MDSCs (P-MDSCs) which have partly overlapping functions^8,16^. MDSC-driven suppressive activities are characterized by the inhibition of CD4 + and CD8 + T cell activation and function, driving and recruiting T regulatory cells, and the production of inhibitory cytokines such as TGF-β^8,17,18^. The numbers of peripheral blood MDSCs are increased in several diseases. For example they are elevated in parasitic, fungal, bacterial and viral diseases^19^ and cancers such as ovarian, breast, melanoma, and gastric^16,20^. It remains problematic to understand why these cells appear in the blood of COVID-19 patients and what their role is, if any, during COVID-19 infections. We hypothesized that there is an association between the frequency of blood MDSCs with COVID-19 disease severity and their ability to suppress T cell proliferation via TGF-β and FOXP3–dependent mechanisms. Thus, serum TGF-β levels and functionality of these cells in COVID-19 patients was evaluated.

## Materials and methods

### Study design and participants

26 COVID-19 patients’ including14 severe patients from the intensive care unit (ICU) and 12 moderate patients from an outpatient clinic were enrolled in the study. In addition, 11 healthy volunteer HC were enrolled from December 2021 to February 2022.

All patients were PCR positive for SARS-CoV-2 infection using a nasal and pharyngeal swab specimens (NPS) test according to the WHO guidance^21^. Patients were diagnosed according to the national guidelines for the new 2019 corona virus and hospital management guidelines against coronavirus as described before^22^.

Exclusion criteria in this study was hematological diseases like chronic myeloid leukemia (CML) or chronic lymphocyte leukemia (CLL), acquired immunodeficiency syndrome (AIDS), Tuberculosis (TB), Influenza, blood transfusion individuals and use of immunosuppressive drugs. This study was approved by ethics committee of Shahid Beheshti Medical University with ID number IR.SBMU.MSP.REC.1400.445, issued by the Institutional Review Board for human studies of the Masih Daneshvari Hospital, Tehran, Iran in October 2021.

### Isolation of peripheral blood mononuclear cells (PBMCs)

Ten ml of whole blood was collected in tubes containing ethylene-diamine-tetra-acetic acid (EDTA) as an anticoagulation from HCs and patients after obtaining informed consent. PBMC were separated based on density by centrifugation over lymphoprep (Ficoll method) with a specific density of 1.076 g/mL as described before^23^ Two ml whole blood harvested in tubes without anti-coagulants in order to determine serum TGF-β levels by ELISA (R&D Systems, Minneapolis, MN, USA). Medical records were reviewed for clinical history, laboratory analyses, previous diseases and comorbidities.

### Isolation of MDSCs

MDSCs (HLA-DR-CD33 + CD11b +) were purified from only freshly isolated PBMCs of COVID-19 patients following magnetic separation with human anti-HLA-DR, CD33 and CD11b microbeads (Miltenyi-Biotec, Bergisch Gladbach, Germany) according to the Manufacturer’s instructions^24^. Briefly, HLA-DR^+^ cells were depleted using anti–HLA-DR microbeads and then CD33^+^ CD11b^+^ cells were positively selected using CD33 and CD11b microbeads from the HLA-DR- fraction. Approximately 0.3 × 10^6^ MDSCs were obtained from 25 to 30 × 10^6^ PBMCs, with a viability of g > 90% and a purity of > 85%. MDSCs were quantified as a percentage of total MDSCs. A cutoff of < 3% MDSC was used to define the upper limit of normal of the MDSC proportion based on previously published data demonstrating that healthy donors have < 2% MDSC of circulating PBMC^25^.

### Flow cytometry analysis

The antibodies used for cell surface and intracellular cell staining are shown in Table 1. For cell surface staining, cells were suspended in a tube containing FACS buffer (PBS 1x, Sodium azide 0.01% and BSA 1%) and surface staining was measured following incubation with the antibody for 30 min at 4 °C. Following surface staining, cells were fixed and permeabilized (BD Biosciences, San Diego, USA) at 4 °C for 15 min to enable intracellular staining. The cells were washed with cold PBS and intracellular staining was performed at 4 °C for 30 min. Cells were then washed with cold PBS and then 1 × 10^4^ events were counted and then the data was analyzed by FlowJo Software version 10 (BD Biosciences).

**Table 1:** Antibodies used in flow cytometry.

**Surface markers**			
mAB	Clone	Manufacture	Isotype
HLA-DR-APC	Tü36	BioLegend, San Diego, USA	Mouse IgG2b, k
CD11b-FITC	94	Beckman coulter	Mouse, IgM
CD14-PerCP-Cy5.5	Mφp9	BD Biosciences, USA	Mouse, IgG2b,k
CD15-PE	H198	eBioscience, San Diego, USA	Mouse, IgM,k
CD4-PE	RPA-T4	Immunostep, Salamanca, Spain	Mouse IgG1
CD8-APC	SK1	BioLegend, San Diego, USA	Mouse,IgG1,k
CD25-PE-Cy5.5	PC61.5	eBioscience, San Diego, USA	Rat/IgG1, lambda
**Intracellular markers**			
mAB	Clone	Manufacture	Isotype
Foxp3-APC	236A/E7	eBioscience, San Diego, USA	Mouse, IgG1,k
IFN-γ-PerCP	XMG1.2	BioLegend, San Diego, USA	Rat, IgG1, k
TGF-β-PE	TW4-9E7 (RUO)	BD Biosciences, USA	Mouse, IgG1,k

### Co-culture of MDCS with PBMC

Isolated MDSCs were co-cultured with carboxyfluorescein succinimidyl ester (CFSE)-labeled-autologous MDSC-depleted PBMCs (MDPs) from patients or with CFSE-labeled-homologous PBMCs from HCs for proliferation assays at a ratio of 1:2 and 1:5, as described previously^26^. The MDP population included lymphocytes (70–90%, T cells—70–85%, B cells—5–10% and NK cells—5–20%), monocytes (10–20%) and dendritic cells (1–2%)^27^.

Proliferation was induced by cell activation with phytohemagglutinin (PHA) (2.5 μg/mL; Gibco, Thermo Fisher Scientific, Waltham, MA, USA) for 5 days at 37 °C in RPMI 1640 medium with 10% FCS, 5 mM l-glutamine, and 100 U/mL penicillin and streptomycin (all from Invitrogen, Thermo Fisher Scientific, Inc.) as explained before^28^. In order to detect intracellular expression of IFN-γ, Foxp3 and TGF-β, autologous MDP and homologous PBMCs were co-cultured, at a ratio of 1:2, in the presence of PHA (2.5 μg/mL) for 3 days^29,30^. Cells were then centrifuged at 1100xg and the supernatants collected and stored at − 20 °C until used for detecting TGF-β. The cell pellets were washed and then used for immunophenotyping by cell surface and intracellular markers (see above).

### Quantification of TGF-β

The concentration of TGF-β in serum of participants and supernatants of co-cultured cells was measured using enzyme linked immunosorbent assay (ELISA) kit (R&D SYSTEM, Minneapolis, MN, US) according to the manufacturer’s instruction.

### Statistical analysis

Statistical analyses were performed using GraphPad Prism software (Version 8.0.2). Normally distributed data are presented as percentages or mean SD and non-normally distributed data are presented as the median (interquartile range, IQR). Comparisons between groups performed using a parametric (ANOVA) and nonparametric (Kruskal–Wallis test) tests followed by the Tukey's or Dunn's tests, respectively. The correlation between variables was analyzed using a correlation matrix test.

### Ethical approval

This study was approved by ethics committee of Shahid Beheshti Medical University with ID number IR.SBMU.MSP.REC.1400.445, issued by the Institutional Review Board for human studies of the Masih Daneshvari Hospital, Tehran, Iran in October 2021.

## Results

### Study participant characteristics

The demographic and clinical characteristics of the participants: 26 adult patients and 11 HCs are shown in Table 2. The distribution of age varied significantly according to the disease severity (P < 0.03). Patients hospitalized for severe COVID-19 disease had significantly more comorbidities such as autoimmune hepatitis (AIH, n = 2), diabetes mellitus (DM, n = 2), Crohn's disease (n = 1) and cancer (n = 2) (all P < 0.03) and were at a high risk of infection with pseudomonas aeruginosa, candida albicans and mucormycetes (all P < 0.02) compared to moderate cases. One patient with severe disease was infected with CMV and fungi. Moreover, there was a positive correlation between the frequencies of circulating MDSC with autoimmune disease (R = 0.6, P = 0.01), cancer (R = 0.6, P = 0.02) and infections (R = 0.5, P = 0.04) in severe patients.

**Table 2:** Demographic and clinical characteristics of participant’s patients.

	Control	Moderate	Severe	P value
N (%)	11 (29.7)	12 (32.4)	14 (37.8)	
Age in year, mean (range)	38.2 (28–78)	45.1 (21–69)	57.6 (28–88)	P < 0.04
Male, n (%)	6(54.4)	7(58.3)	9(64.2)	
Female, n (%)	5(45.4)	5(41.6)	5(35.7)	
**Comorbidities**				
Autoimmune diseases, n (%)	0 (0)	1 (8.3)	5 (35.7)	P = 0.03
Cancer, n (%)	0 (0)	0 (0)	2 (14.2)	P = 0.03
Infection, n (%)	0 (0)	0 (0)	8 (57.1)	P = 0.02
**Laboratory analyses**				
WBC, Mean (10^9^ cell/L)	7.01	7.20	11.62	P = 0.08
Neutrophils, Mean (10^9^ cell/L)	4.01	5.10	10.03	P = 0.002
Monocytes, Mean (10^9^ cell/L)	0.4	0.7	0.8	P = 0.01
Lymphocytes, Mean (10^9^ cell/L)	2.01	1.6	0.93	P = 0.001
O2 Saturation, mean (range)	98 (97–98)	91.41 (87–94)	18.82 (16.3–28)	P = 0.001
Ct value, mean (range)	35.10 (33.6–40)	22.17 (18.51–28.43)	20.97 (17.41–28.43)	P = 0.01

*Lymphocyte count, Ct value; WBC and neutrophil, counts at the time point of the lowest lymphocyte count. Normal range: WBC 3.5 × 10^9^/L to 8.8 × 10^9^/L, lymphocytes 1.1 × 10^9^/L to 3.5 × 10^9^/L, neutrophils 1.6 × 10^9^/L to 5.9 × 10^9^/L.

In addition, TGF-β levels in 46% (6/ 13) of severe patients who had at least a one chest CT scan before hospitalization (Supplementary Fig. 1 and Table 3) show a correlation with lung fibrosis (P < 0.02). CT scans were performed together with pulmonary functional test (PFT) in some patients and plethysmography. None of the patients had any previous evidence for lung fibrosis.

**Table 3:** Demographic characteristics of COVID-19 patients with lung fibrosis.

	P8	P16	P17	P19	P22	P23
Age (year)	42	28	88	40		58
Sex	Female	Female	Male	Male	Male	Male
Severity of COVID-19	Severe	Severe	Severe (Long-COVID)	Severe	Severe	Severe (Long-COVID)
Alive/dead	Alive	Alive	Alive	Alive	Alive	Dead
TGF-β (pg/ml)	421	377	439	256	253	330

### Sex differences in MDSCs frequency

**Figure 1:**
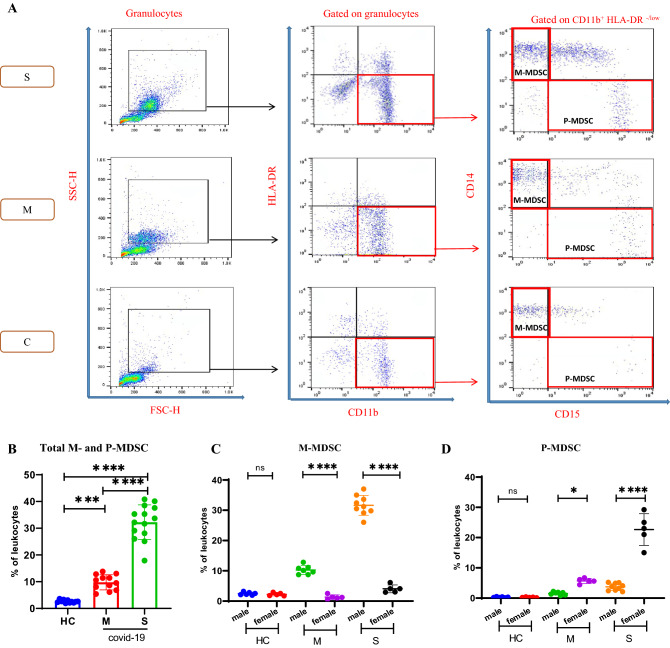
Analysis of circulating myeloid-derived suppressor cells (MDSCs) frequencies in patients with COVID-19. (**A**) Peripheral blood mononuclear cells (PBMCs) obtained from COVID-19 patients, healthy controls (HCs) were used to analyze MDSCs by flow cytometry. Representative dot plots of monocytic (M)-MDSCs (HLA-DR^+/low^CD11b^+^CD14^+^CD15^-^ cells) and polymorphonuclear (P)-MDSCs (HLA-DR^+/low^CD11b^+^CD14^-^CD15^+^ cells) identified using a gating strategy are shown in patients (severe and moderate) and control groups. (**B**) The frequencies of M- and P-MDSCs in the PBMC presented. (**C,D**) Sex differences in the frequencies of M-MDSCs and P-MDSCs between HC and moderate and severe COVID-19 patients. M-MDSCs were increased in both SMPs and MMPs, while P-MDSCs were increased in SFPs and MFPs. All values are presented as the mean or median and 5–95% percentile and comparisons made between control and patient groups were performed using ANOVA or kruskalwalis followed by a the Tukey's or Dunn's tests for normally distributed or non-normally distributed data respectively *P < 0.01, **P < 0.001, ***P < 0.0001 and ****P < 0.0001. *HLA-DR* human leukocyte antigen-D–related, *HC* healthy control, *M* moderate, *S* severe.

M-MDSC are characterized as HLA-DR-/lowCD11b + CD14 + CD15-, and P-MDSC as HLA-DR-/lowCD11b + CD14-CD15 +. Because of the limitation in the number of channels in the FACS instrument, the CD15 marker was not always included; therefore, we named non-M-MDSC the subpopulation HLA-DR-/lowCD11b + CD14-^31,32^. As depicted in the representative panels in Fig. 1A, the gating strategy adopted here identified M-MDSCs and P-MDSCs from the peripheral blood of patients with severe and moderate COVID-19. There was an increase in the frequencies of total circulating MDSCs with disease severity (Fig. 1B). We found similar results whether gating on leukocytes or all granulocytes.

Sex differences in MDSC frequency in COVID-19 patients were detected. M-MDSCs were increased in both severe male patients (SMPs) and moderate male patients (MMPs) compared with severe female patients (SFPs) and moderate female patients (MFPs) (Fig. 1C P < 0.0001 for both). In contrast, P-MDSCs were increased in SFPs and MFPs compared with SMPs and MMPs (Fig. 1D P < 0.0001 and P = 0.0157, respectively). When the results were compared by severity in males and females independently, there was a significant increase in M-MDSCs with increasing severity of COVID-19 in both males (Supplementary Fig. 2A, P < 0.0001) and females (Supplementary Fig. 2B, P = 0.0061) although the effect was much greater in males. In a similar manner, there was a significant increase in P-MDSCs with increasing severity of COVID-19 in both males (Supplementary Fig. 2C, P < 0.0001) and females (Supplementary Fig. 2D, P < 0.0001) although the effect was much greater in males.

### Up regulation of TGF-β^+^ in MDSCs

**Figure 2:**
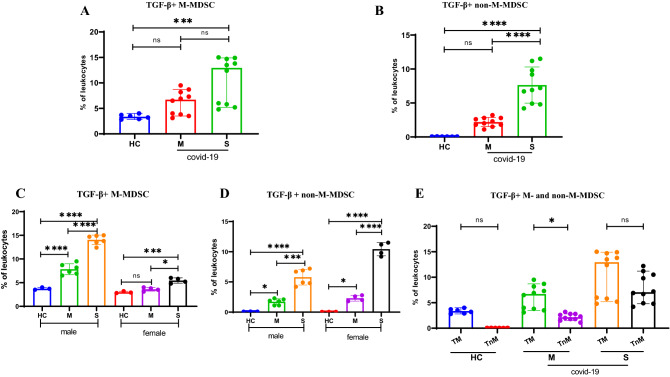
Expression levels of intracellular TGF-β in MDSCs. The frequencies of TGF-β^+^ M-MDSCs (**A**) and TGF-β^+^ non-M-MDSCs (**B**) as the percentage of the CD11b^+^HLA-DR^-/low^ cells in moderate (M) and severe (S) COVID-19 patients compared with healthy control subjects (HC). The effect of sex on the percentage of TGF-β^+^ M-MDSCs (**C**) and of TGF-β^+^ non-M-MDSCs (**D**) is also shown. (**E**) Frequency of TGF-β^+^ M-MDSCs compared to non-M-MDSCs in HC and COVID-19 patients (both M and S). All values are presented as the mean or median and 5–95% percentile and comparisons made between control and patient groups were performed using ANOVA or Kruskal–Wallis followed by a the Tukey’s or Dunn’s tests for normally distributed or non-normally distributed data respectively.*P < 0.01, **P < 0.001, ***P < 0.0001 and ****P < 0.0001. *HC* healthy control, *M* moderate, *S* severe.

To examine the expression of inhibitory mediators such as TGF-β as a readout of MDSC activity, intracellular TGF-β expression levels were evaluated. The gating strategy adopted to identify TGF-β + MDSCs with representative FACs plots is depicted in Supplementary Fig. 3. The intracellular levels of TGF-β in M-MDSCs were higher in severe patients compared to HC subjects (Fig. 2A P = 0.0009). There was no significant difference between intracellular TGF-β levels between moderate and severe COVID-19 patients (P = 0.2, Fig. 2A). In contrast, intracellular TGF-β expression in non-M-MDSCs (HLA-DR^-^/low CD11b^+^CD14^-^TGF-β^+^ cells) significantly higher in severe COVID-19 patients compared with both moderate COVID-19 and HC groups (Fig. 2B, all P < 0.0001).

Percentages of TGF-β + M-MDSCs in HC were similar in males and females. However, the frequency of TGF-β + M-MDSCs in COVID-19 patients was elevated in a much greater step-wise manner in males compared to that seen in females (Fig. 2C). In contrast, the step-wise increase in the frequency of TGF-β + non-M-MDSCs in female COVID-19 patients compared to HC was greater than that observed in male patients (Fig. 2D). Furthermore, M-MDSCs show a higher intracellular expression of intracellular TGF-β than non M-MDSCs in moderate group (Fig. 2E; P = 0.0430) but not in severe and HC groups (Fig. 2E).

### Isolated MDSCs suppressed proliferation and IFN-γ production of autologous and homologous T cells

**Figure 3:**
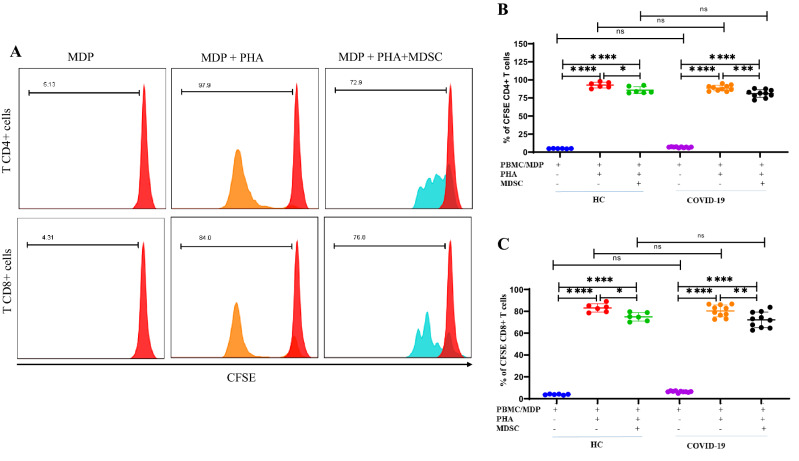
Isolated MDSCs, suppressed CD4 + and CD8 + T cells response in vitro. MDSCs isolated from fresh PBMC of the patients co-cultured with CFSE labeled-autologous MDPs from the patients and CFSE labeled-homologous PBMCs from HCs in the presence of PHA for 5 days. (**A**) Histograms show representative CD4^+^ and CD8^+^ T cell proliferation as assessed by CFSE dilution and flow cytometry. The numbers in the plots indicate the frequency of proliferating T cells. The unstimulated MDPs are indicated in red, PHA-stimulated MDPs are indicated in orange and PHA-stimulated MDPs in presence of MDSCs are indicated in blue. (**B,C**) Dot plots show the percentage of proliferating CD4^+^ and CD8^+^ T cells from 10 COVID-19 patients and 6 HCs. All values are presented as the mean and 5–95% percentile and comparisons made between control and patient groups were performed using ANOVA followed by a Tukey’s test. *P < 0.01, **P < 0.001, ***P < 0.0001 and ****P < 0.0001. Abbreviations: MDP: MDSC depleted PBMC.

MDSCs were isolated from COVID-19 patients and subsequently co-cultured with autologous MDP and homologous PBMCs as source of T cells and T cell proliferation was measured. In initial experiments, PBMCs gave similar results to purified leukocytes (data not shown). Cells cultured at a ratio of 1:5 did not show suppression of proliferation and so data are shown for the results of cell cultures at a ratio of 1:2. Representative flow cytometry plots of MDSC purity and cytology by microscopy are shown in Supplementary Fig. 4A,B. Supplementary Figure 5 also shows representative flow cytometry plots of T cell proliferation in vitro. We co-cultured HC CFSE-labeled PBMC (homologous) or CSFE-labeled MDP (autologous) with patients’ MDSC to compare the T cells response in COVID-19 patients with HCs. As shown in Fig. 3A, PHA induced strong T cell proliferation. However, in presence of MDSCs, there was a small but significant suppression of both CD4 + (Fig. 3B) and CD8 + (Fig. 3C) T cell proliferation.

**Figure 4:**
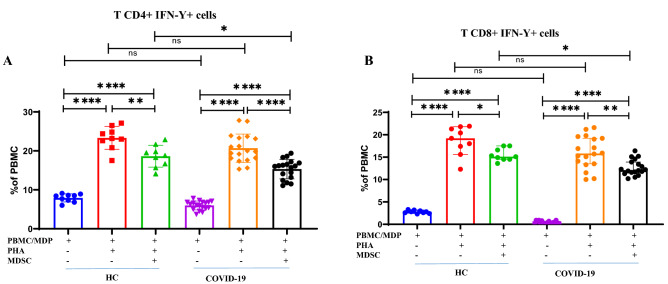
Isolated MDSCs decreased intracellular IFN-γ in T cells. Isolated MDSCs from COVID-19 patients were co-cultured with autologous MDPs from the patients and homologous PBMCs from HCs in the presence of PHA for 3 days. (**A,B**) Dot plots show the percentage of CD4 + IFN-γ + T cells from 18 COVID-19 patients and 9 HCs. All values are presented as the mean and 5–95% percentile and comparisons made between control and patient groups were performed using ANOVA followed by a Tukey’s test. *P < 0.01, **P < 0.001, ***P < 0.0001 and ****P < 0.0001. *HC* healthy control, *P* patients, *MDP* MDSC depleted PBMC.

As expected, PHA induced high expression levels of intracellular IFN-γ in CD4 + and CD8 + T cells whilst co-culture with MDSCs from COVID-19 patients significantly suppressed intracellular IFN-γ (Supplementary Fig. 6). Incubation of MDSCs from COVID-19 patients with either autologous CD4 + T cells or autologous CD8 + T cells gave a more significant reduction in the level of intracellular IFN-γ expression than observed with homologous CD4 + and CD8 + T cells (Fig. 4A P = 0.02 and Fig. 4B P = 0.03, respectively) despite similar levels of induction.

### COVID-19 MDSCs suppress CD4 + CD25 + Foxp3 + cells and evoke CD4 + CD25-Foxp3 + expansion

**Figure 5:**
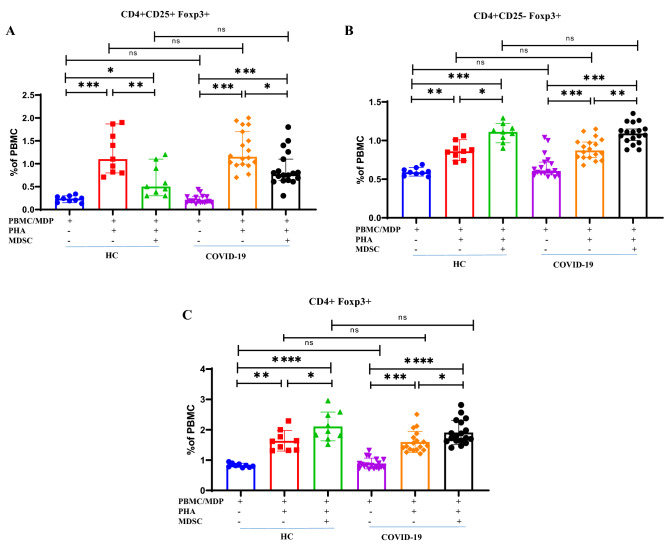
Isolated MDSCs induced CD4 + CD25- Foxp3 + T cells. Isolated MDSCs from fresh PBMC of patients co-cultured with autologous MDPs from the same patients and homologous PBMCs from HCs in the presence of PHA for 3 days. Dot plots show the percentage of CD4^+^CD25^+^Foxp3^+^ (**A**), CD4^+^CD25^-^Foxp3^+^ (**B**) and CD4^+^Foxp3^+^ cells (**C**) from 18 COVID-19 patients and 9 HCs All values are presented as the median and 5–95% percentile and comparisons made between control and patient groups were performed using kruskalwalis followed a Dunn's test. *P < 0.01, **P < 0.001, ***P < 0.0001 and ****P < 0.0001. *HC* healthy control, *P*: patients, *MDP* MDSC depleted PBMC.

TGF-β promotes the expansion of CD4 + CD25 + Foxp3 + regulatory T cells in vitro and in vivo^33^. Thus, we assessed the impact of MDSCs on CD4 + CD25 + Foxp3 + T in HC and COVID-19 patients. Representative FACS plots are shown in Supplementary Fig. 7 indicating the effect of co-culturing MDSCs with autologous MDP and homologous PBMCs on CD4 + CD25 + Foxp3 + cell frequency. Co-culture of autologous MDSCs, significantly suppressed the expansion of PHA-stimulated CD4 + CD25 + Foxp3 + T cells (Fig. 5A). No significant difference was seen on the frequency of CD4^+^CD25^+^Foxp3^+^ T cells in autologous and homologous co-cultures (Fig. 5A P = 0.9).

In contrast, the PHA-induced proliferation and expansion of CD4^+^CD25^-^Foxp3^+^ regulatory precursor T cells was significantly enhanced by co-culture with MDSCs from both patients and HCs (Fig. 5B P = 0.002, P = 0.03, respectively). No differences in the increase in CD4^+^CD25^-^Foxp3^+^ T cell frequencies were observed in response to culture with autologous and homologous co-cultures (Fig. 5B P = 0.83). Regardless of CD25 expression, the number of CD4^+^Foxp3^+^ T cells in co-culture with MDSCs was also considerably elevated in both patients and HCs (Fig. 5C P = 0.02, P = 0.04, respectively).

### Serum TGF-β is elevated in patients with COVID-19

**Figure 6:**
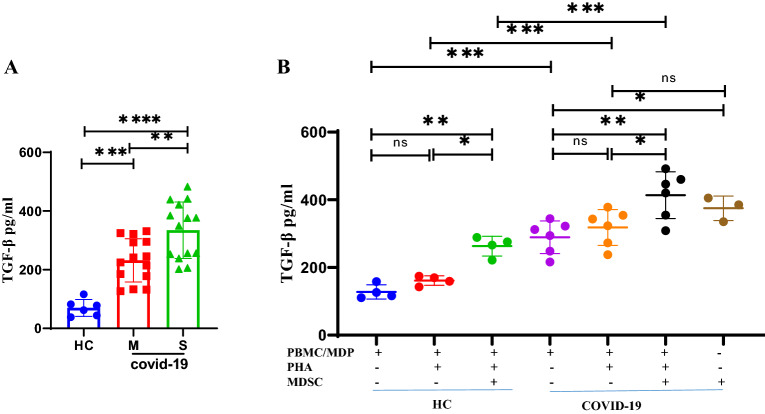
Levels of TGF-β in serum from COVID-19 patients and Healthy control subjects (HCs). (**A**) Serum TGF-β was measured in 28 COVID-19 patients (14 moderate and 14 severe) and 6 HCs. (**B**) Release of TGF-β into the media of MDSCs isolated from fresh peripheral blood cells of patients co-cultured with autologous MDPs from the same patients and homologous PBMCs from HCs in the presence of PHA for 3 days. All values are presented as the mean and 5–95% percentile and comparisons made between control and patient groups were performed using ANOVA followed by a Tukey's test**.** *P < 0.01, **P < 0.001, ***P < 0.0001 and ****P < 0.0001. *HC* healthy control, *M* moderate, *S* severe, *MDP* MDSC depleted PBMC, *MHC* male healthy control, *MMP* moderate male patients, *SMP* severe male patients, *FHC* female healthy control, *MFP* moderate female patients, *SFP* severe female patients.

Serum concentrations of TGF-β in COVID-19 patients and HCs were measured by ELISA. Patients with severe disease had significantly elevated serum levels of TGF-β compared with patients with moderate COVID-19 and HCs (P = 0.003, P < 0.0001, respectively). Patients with moderate COVID-19 also demonstrated significantly elevated levels of TGF-β compared to HC (P = 0.0001) (Fig. 6A).

### TGF-β release from co-cultured MDSCs and PBMC

PHA had no significant effect on the release of TGF-β from autologous MDPs and homologous PBMCs alone. There was a significant difference in the levels of TGF-β detected between co-cultures of MDSCs with autologous MDPs and homologous PBMCs (Fig. 6B P = 0.0001). The concentrations of TGF-β were significantly higher following co-culture of autologous MDP (P = 0.02) and homologous PBMCs (P = 0.01) with MDSCs (Fig. 6B). PBMCs from HCs release less TGF-β than MDP from patients in the presence of MDSCs (Fig. 6B P = 0.0007). Importantly, we found high levels of TGF-β in MDSCs alone compared to the autologous MDPs (Fig. 6B P = 0.03).

## Discussion

In this study we show a high percentage of M-MDSCs in males and of P-MDSCs in female patients with severe and moderate COVID-19 particularly in the more severe patients who required intensive care. In addition, increased serum levels of TGF-β and of MDSCs were observed in COVID-19 patients with a significant correlation between severity and serum TGF-β levels. Most importantly, this current study revealed a correlation between elevated serum levels of TGF-β and lung fibrosis in severe COVID-19 patients. Isolated M-MDSCs produced higher levels of intracellular TGF-β than non-M-MDSCs. Purified MDSCs from COVID-19 patients not only caused suppression of both CD4 + and CD8 + T cell proliferation and IFN-γ production, but also induced the production of TGF-β. We used patients' MDSC to co-culture with HC CFSE-labelled PBMC (homologous) or with CSFE-labelled MDP (autologous) to avoid potential problems using only autologous T cells (MDPs) due to the expected low response in COVID-19 patients. In addition, MDSCs expanded the CD4 + Foxp3 + CD25^-^ precursor regulatory T cell population in co-culture whilst suppressing that of the mature regulatory CD4 + Foxp3 + CD25^+^ T cell population.

Studies show a discrepancy in relation to MDSCs numbers and the severity of various diseases. For example, M-MDSCs are increased in the peripheral blood of COVID-19 patients and predict the severity of the disease^26^ or poor outcome^34^. In contrast, increased percentages of P-MDSCs are detected in severe and mild COVID-19 patients^14,35^. Although one study showed increased P-MDSCs in severe, but not mild or moderate patients with COVID-19^36^.

There is no published mechanism that may account for the upregulation of the different subtypes of MDSCs in male and female patients with COVID-19. However, previous data also shows a variation between sexes in severe patients^37,38^. Some evidence suggest that this phenomenon is disease-dependent as a higher frequency of P-MDSCs is observed in females with viral myocarditis^39^ and in male mice with liver metastases^40^. Furthermore, M-MDSCs are predominant in males with glioblastoma whilst P-MDSCs are higher in female patients^41^. Dong and co-workers found that compared with male patients with systemic lupus erythematosus (SLE), female patients had a higher frequency of MDSCs and that this may be due to 17β-estradiol-induced accumulation of MDSCs in an SLE mouse model^42^. Furthermore, Köstlin-Gille and colleagues showed that the levels of P-MDSCs in breast milk of lactating mothers is influenced by the child’s sex with significantly higher levels found in breast milk given to daughters compared to sons^43^. Moreover we measured the frequency of MDSCs in isolated PBMC not from whole blood. This contrasts with our previous analysis of MDSC frequencies in the whole blood of Iranian COVID-19 patients^44^. Comparison of the two data sets indicates that PBMC isolation has effect on the frequency of different types of MDSCs, at least in these studies.

In-vitro and in-vivo studies indicating higher suppressive activity of M-MDSCs than P-MDSCs^45–47^. Interestingly, the current data shows a higher frequency of M-MDSCs in males and based on the suppressive nature of M-MDSCs, the higher susceptibility of males compared to females for COVID-19 may explain, at least in part, this scenario^26,48^. However, due to the number of participants in our study we were not able to formally address whether these effects were a consequence of severity. However, the COVID-19 patients with more severe disease were predominantly male and older than female patients who had less severe disease. Moreover, there was also a correlation between the male sex and the occurrence of comorbidities.

Evidence suggests that the behavior of MDSCs during infection depends upon the type and the virulence mechanisms of the invading pathogen, the disease stage and the infection-related pathology^49^. While the immunosuppressive characteristic of MDSCs may help to preserve tissue homeostasis and prevent hyperinflammation at early stages of the infection^14^, attenuation of an efficient immune response in latter stages may have significant pathogenic effects on severe COVID-19^50^. Activated MDSCs inhibit the function of NK, CD4 + and CD8 + T cells through L-arginine depletion following enhanced arginase production. Moreover they contribute to the cytokine storm by releasing high amounts of proinflammatory cytokines during COVID-19 progression^51,52^. Furthermore, MDSCs suppress the proliferation of allogeneic and autologous T cells via the release of TGF-β^53^. In this study, increased serum TGF-β levels and levels of TGF-β in the supernatant of co-cultures of MDSCs with T cells were demonstrated. This is in agreement with the observation that IFN-γ production upon SARS-CoV-2 peptide stimulation was inhibited by P-MDSCs via TGF-β- and iNOS-mediated pathways^34^. Since we observed intracellular TGF-β in MDSCs and in the supernatants of cells in co-culture, we speculate that serum levels of TGF-β are, at least in part, due to its release from MDSCs.

TGF-β is produced by immune and non-immune cells and plays a diverse set of roles in cell differentiation and tissue repair^54,55^. Moreover, TGF-β is important in the progression of lung fibrosis^56^. Fibroblasts and airway smooth muscle cells (ASMCs) can activate TGF-β production through integrin binding, resulting in proliferation and tissue fibrosis in multiple solid organs^57–59^. The current data suggest that MDSCs-elicited TGF-β may have a link with fibrosis that needs to be examined in future focused studies. Elevated serum levels of TGF-β may, therefore, be considered as a predictive factor for the outcome of lung fibrosis^60^, or as a prognostic factor for the diagnosis of COVID-19.

There are conflicting data regarding the frequency of regulatory T cells (Tregs) during SARS-CoV-2 infection with reports indicating both decreased^61^ and increased^62,63^ frequency of CD4^+^CD25^+^Foxp3^+^ Treg cells. M-MDSCs can expand Foxp3^+^ Tregs in vivo^64^, but a direct link between the COVID-19-induced MDSCs and Tregs has not been demonstrated yet. In the current study, isolated MDSCs induce the expansion of CD4^+^CD25^-^FoxP3^+^ precursor regulatory T cells but suppress that of mature Tregs. Future studies should increase the duration of co-culture to determine whether this affects the impact on mature T cells. Interestingly, the poor outcome of COVID-19 patients was accompanied by increased serum levels of soluble CD25 (sCD25)^65^ and with high FoxP3 expression in Tregs^62,63^. Future studies should investigate whether the increase in serum CD25 levels correlates with shedding of this receptor by Tregs in COVID-19 patients.

CD4^+^CD25^-^FoxP3^+^ cells were first reported in 2006 in patients with cancer and autoimmune disease^66^. However, their exact mechanism of action in COVID-19 patients remain to be elucidated. Since Tregs play a vital function in diminishing an extreme inflammatory response, they potentially dampen the antiviral response following an intense cytokine storm and contribute to the secondary re-expansion of disease^67^. The differing effects of MDSCs on precursor and mature Tregs requires further study.

The limitations of the current study include that this is a single center study and the lack of a validation group and the relative low subject numbers as this is a pilot study therefore no formal power calculation. However, the results of this exploratory study has led to the identification of novel hypotheses. A deeper analysis of the functional and genetic differences between TGF-β + M-MDSCs vs TGF-β + non-M-MDSCs may provide a broad view of how these cells act in COVID-19.

In summary, our data shows that in COVID-19 patients the numbers of peripheral blood MDSCs are increased which is associated with a worse outcome of disease. Moreover, these cells can suppress stimulated homologous and autologous T cell proliferation possibly via the induction of precursor Tregs and the release of TGF-β. In conclusion, the current data indicate that monitoring of serum MDSCs and TGF-β levels in patients with COVID-19 may be used as diagnostic parameter and may also be used as a tool during therapeutic interventions.

## Supplementary Information

Supplementary Figures.

## Data Availability

The datasets used and/or analysed during the current study available from the corresponding author on reasonable request.
