# Supplementary material for: Immunophenotype and function of circulating myeloid derived suppressor cells in COVID-19 patients
Source: Sci Rep. 2022 Dec 29;12:22570. doi: 10.1038/s41598-022-26943-z (PMC9799710; doi:10.1038/s41598-022-26943-z)
Supplement: Supplementary file 1 — Supplementary Figures. [file 41598_2022_26943_MOESM1_ESM.pptx]

## Slide 1
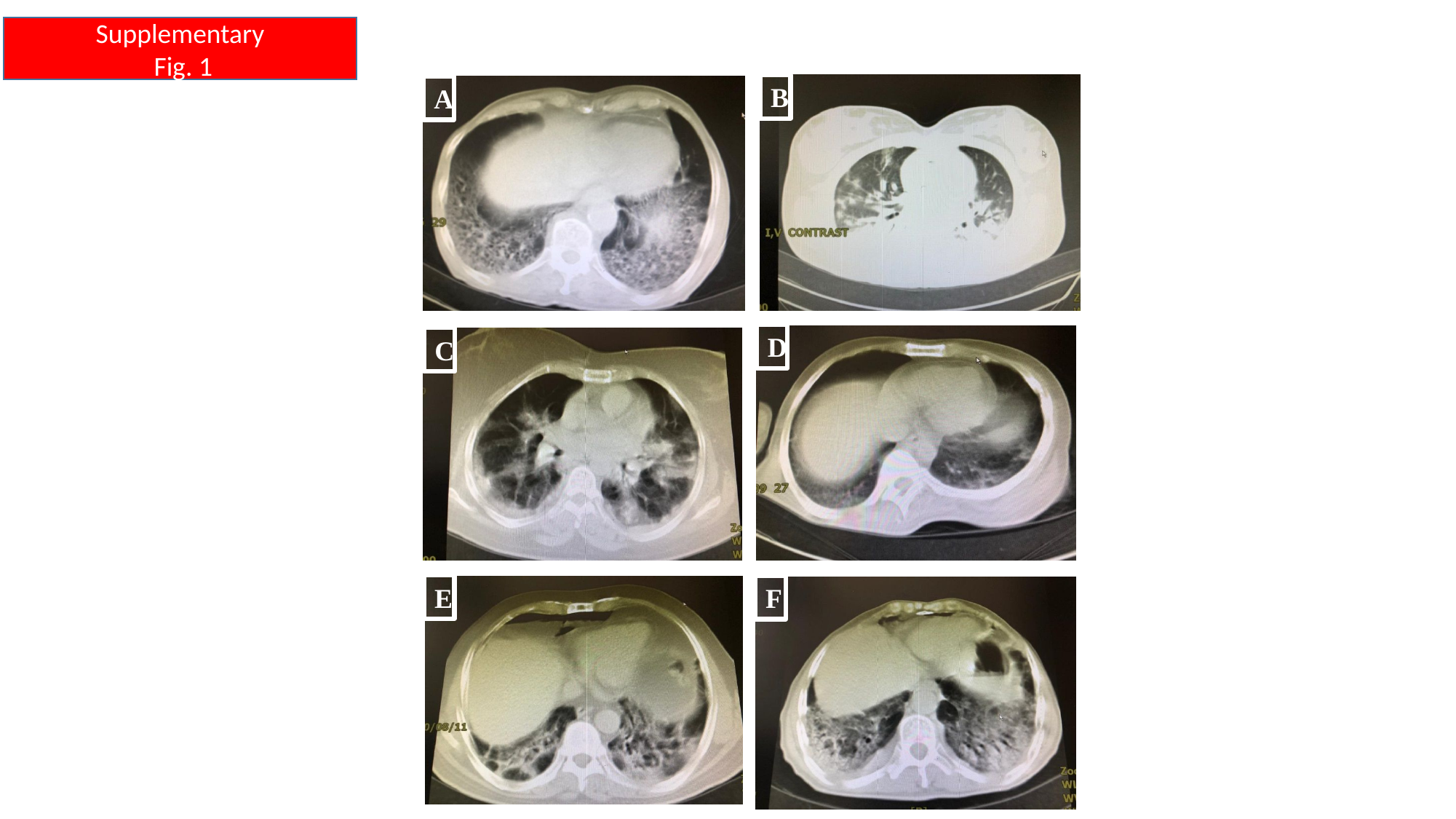

Supplementary
 Fig. 1
B
A
D
C
E
F

## Slide 2
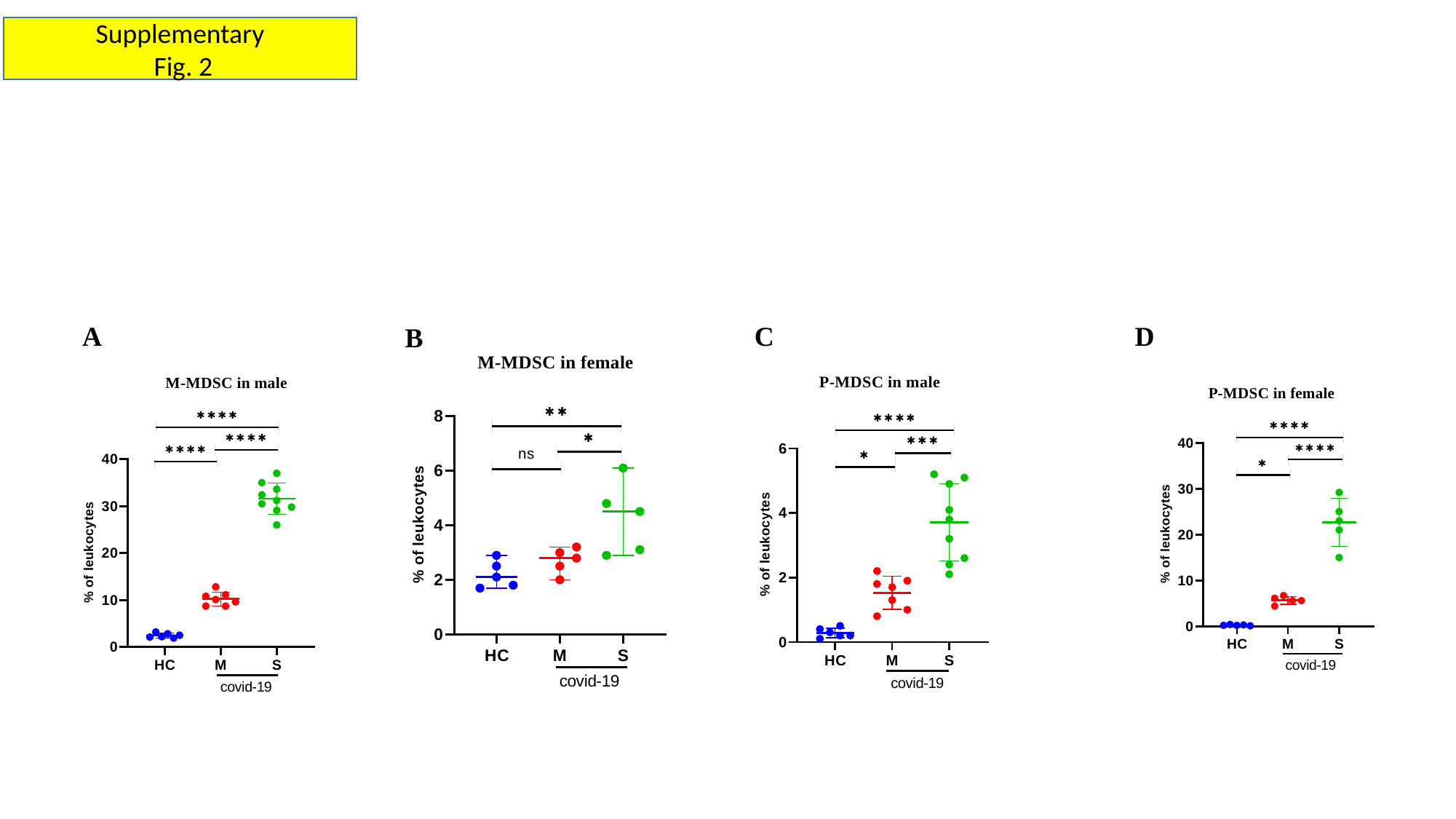

Supplementary
 Fig. 2
A
C
D
B

## Slide 3
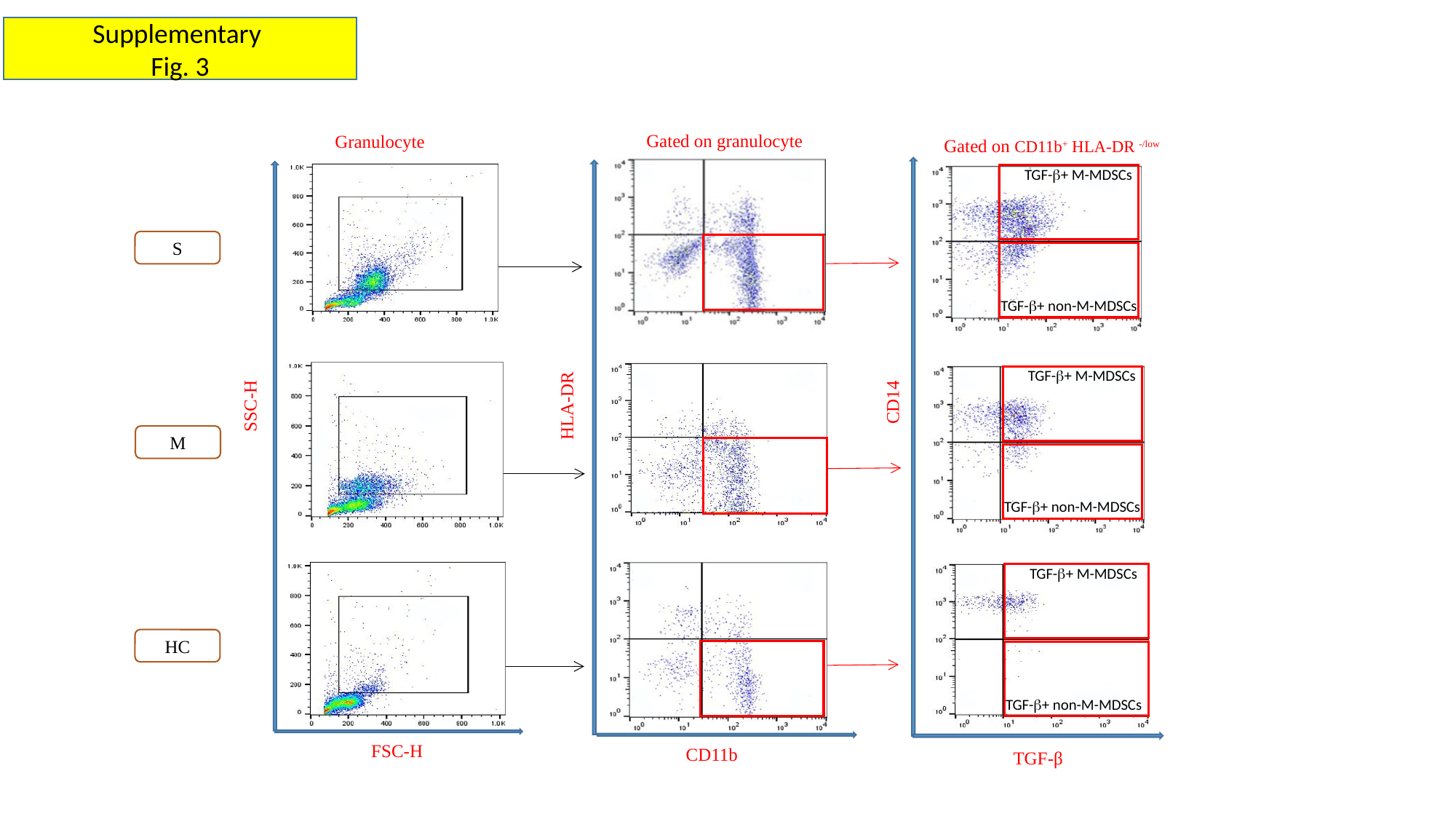

Supplementary
Fig. 3
Gated on granulocyte
Granulocyte
SSC-H
FSC-H
HLA-DR
CD11b
S
M
HC
Gated on CD11b+ HLA-DR -/low
CD14
TGF-β
TGF-β+ M-MDSCs
TGF-β+ non-M-MDSCs
TGF-β+ M-MDSCs
TGF-β+ non-M-MDSCs
TGF-β+ M-MDSCs
TGF-β+ non-M-MDSCs

## Slide 4
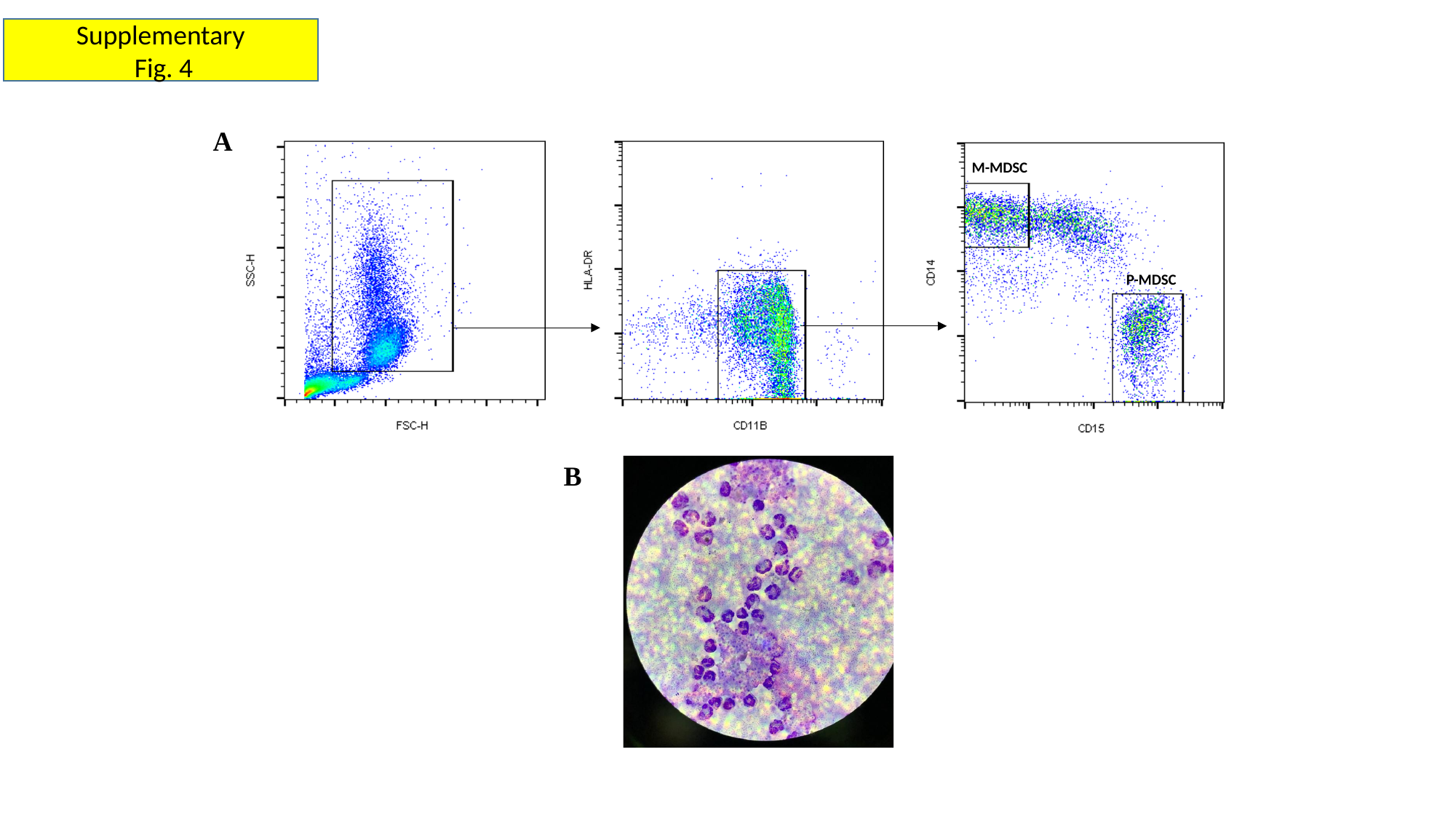

Supplementary
 Fig. 4
A
B
M-MDSC
P-MDSC

## Slide 5
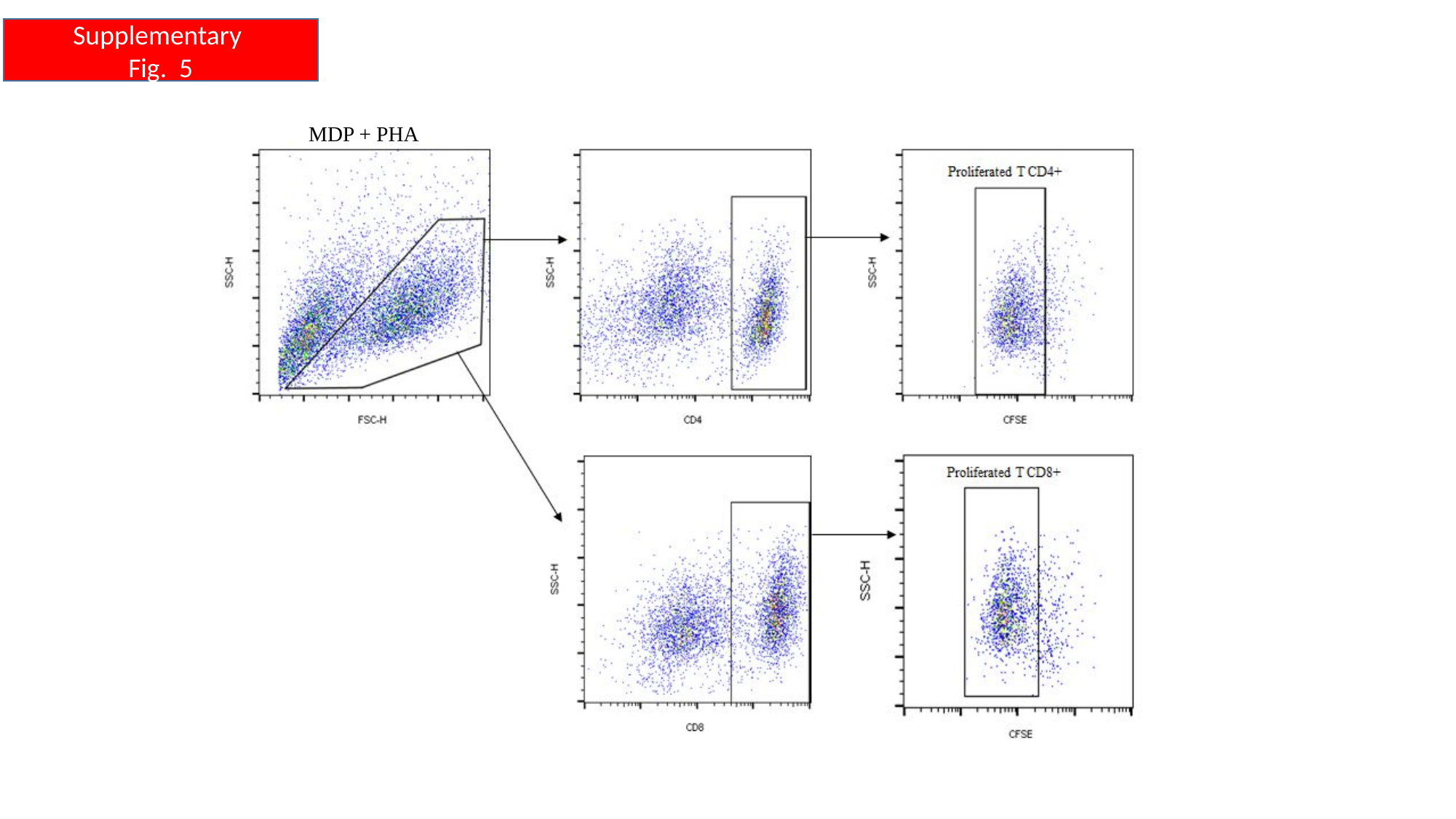

Supplementary
Fig. 5
MDP + PHA

## Slide 6
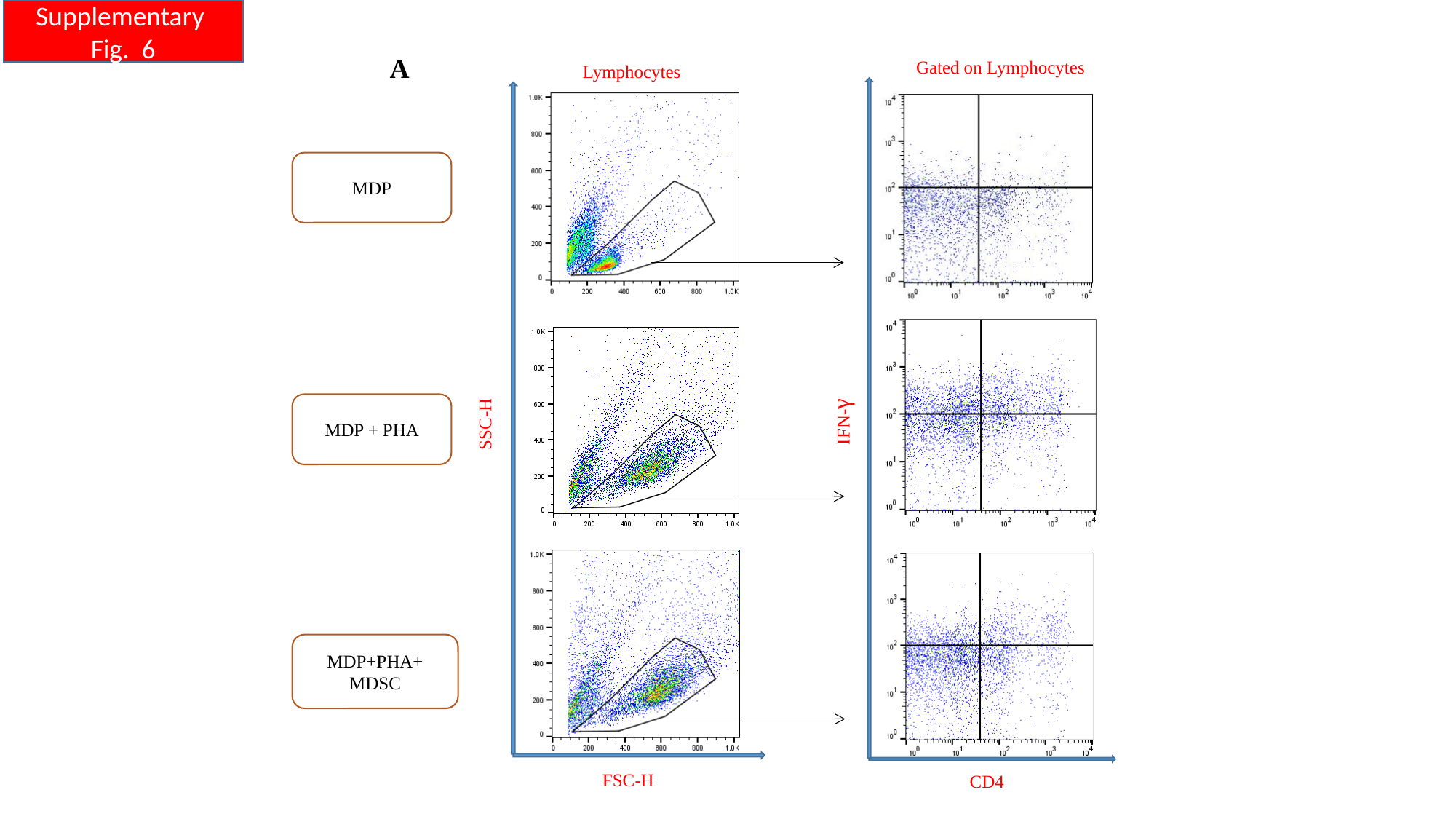

Supplementary
Fig. 6
A
Lymphocytes
SSC-H
FSC-H
MDP
MDP + PHA
MDP+PHA+ MDSC
IFN-γ
CD4
Gated on Lymphocytes

## Slide 7
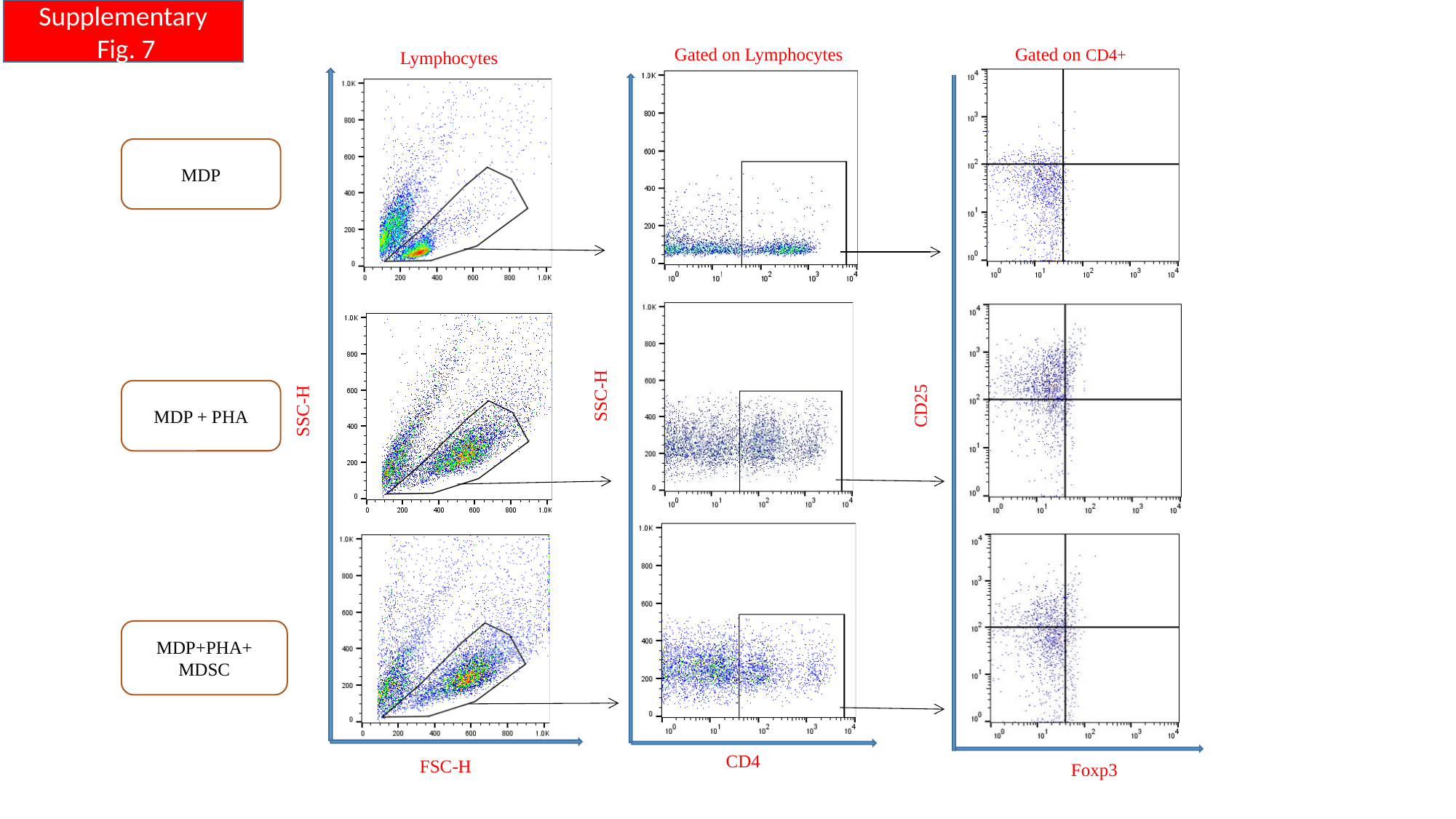

Supplementary
 Fig. 7
Gated on CD4+
Gated on Lymphocytes
Lymphocytes
SSC-H
FSC-H
CD25
Foxp3
SSC-H
CD4
MDP
MDP + PHA
MDP+PHA+ MDSC
